# Interactions between renal vascular resistance and endothelium‐derived hyperpolarization in hypertensive rats in vivo

**DOI:** 10.14814/phy2.14168

**Published:** 2019-07-31

**Authors:** Søs U. Stannov, Jens Christian Brasen, Max Salomonsson, Niels‐Henrik Holstein‐Rathlou, Charlotte M. Sorensen

**Affiliations:** ^1^ Institute of Biomedical Sciences, Heart, Renal and Circulation University of Copenhagen Copenhagen Denmark; ^2^ Department of Electrical Engineering Technical University of Denmark Lyngby Denmark; ^3^ Trelleborg Hospital Trelleborg Sweden

**Keywords:** Hyperpolarization, hypertension, renal, renal vascular resistance

## Abstract

Endothelium derived signaling mechanisms play an important role in regulating vascular tone and endothelial dysfunction is often found in hypertension. Endothelium‐derived hyperpolarization (EDH) plays a significant role in smaller renal arteries and arterioles, but its significance in vivo in hypertension is unresolved. The aim of this study was to characterize the EDH‐induced renal vasodilation in normotensive and hypertensive rats during acute intrarenal infusion of ACh. Our hypothesis was that the increased renal vascular resistance (RVR) found early in hypertension would significantly correlate with reduced EDH‐induced vasodilation. In isoflurane‐anesthetized 12‐week‐old normo‐ and hypertensive rats blood pressure and renal blood flow (RBF) was measured continuously. RBF responses to acute intrarenal ACh infusions were measured before and after inhibition of NO and prostacyclin. Additionally, RVR was decreased or increased using inhibition or activation of adrenergic receptors or by use of papaverine and angiotensin II. Intrarenal infusion of ACh elicited a larger increase in RBF in hypertensive rats compared to normotensive rats suggesting that endothelial dysfunction is not present in 12‐week‐old hypertensive rats. The EDH‐induced renal vasodilation (after inhibition of NO and prostacyclin) was similar between normo‐ and hypertensive rats. Reducing RVR by inhibition of *α*_1_‐adrenergic receptors significantly increased the renal EDH response in hypertensive rats, but a similar increase was found after activating *α*‐adrenergic receptors using norepinephrine. The results show that renal EDH is present and functional in 12‐week‐old normo‐ and hypertensive rats. Interestingly, both activation and inactivation of *α*_1_‐adrenergic receptors elicited an increase in the renal EDH‐induced vasodilation.

## Introduction

Arterial hypertension is associated with increased peripheral resistance and is a major risk factor for cardiovascular complications. There is strong evidence from human and animal studies for a primary role of altered renal control of Na^+^ and water excretion in the pathogenesis of hypertension (Guyton et al., 1972). Renal Na^+^ and water excretion is determined by the balance between renal glomerular filtration rate (GFR) and reabsorption of Na^+^ and water. Control of the renal microcirculation is therefore central for the control of arterial blood pressure.

Endothelium derived signaling mechanisms play an important role in the regulation of vascular tone and endothelial dysfunction is often found in hypertension (Mattei et al., 1997; Cupisti et al., 2000). Endothelial dysfunction may be due to decreased bioavailability of nitric oxide (NO), possibly caused by increased oxidative stress seen in hypertension (Touyz, 2004). NO and prostacyclin are main contributors to endothelium‐dependent vasodilation. Endothelium‐dependent dilation remaining after inhibition of NO and prostaglandin production is by definition caused by endothelium‐derived hyperpolarization (EDH) (Mombouli and Vanhoutte, 1997). The nature of EDH is still not clarified and the relative importance of EDH and other endothelial vasodilatory mechanisms may differ depending on vascular bed, species, age, physiological, and pathophysiological conditions.

Studies on renal EDH in hypertension have generated conflicting results. In afferent arterioles from 13 to 16‐week‐old spontaneously hypertensive rats (SHR) inhibition of NO production reduced afferent arteriolar diameter more compared to arterioles from normotensive rats whereas renal EDH was significantly reduced suggesting a high NO production and low contribution from EDH in young SHR (Ito and Carretero, 1992; Hayashi et al., 1999). However, in renal arteries from 8‐week‐old SHR, EDH‐induced vasodilation is stronger (Bussemaker et al., 2003) but decreases with increasing age (Bussemaker et al., 2003; Michel et al., 2008). Possibly the difference in vessel size and function contributes to this discrepancy as EDH has been shown to be more prevalent in smaller resistance vessels (Shimokawa et al., 1996; Boettcher and Wit, 2011).

In vascular smooth muscle cells (VSMC) from afferent arterioles from normotensive rats a membrane potential (*V*_m_) of −40 mV has been reported (Loutzenhiser et al., 1997). *V*_m_ has not been measured in similar VSMC from hypertensive animals but in renal artery VSMC from 8‐week‐old SHR *V*_m_ is −45 mV. At increasing age and blood pressure the *V*_m_ depolarized to −35 mV at 22 months (Bussemaker et al., 2003). In interlobar VSMC from 15‐week‐old normo‐ and hypertensive rats *V*_m_ is −50 mV and −32 mV respectively (Martens and Gelband, 1996). It seems plausible that at a more depolarized *V*_m_ a stronger hyperpolarizing stimulus is required to attain the same degree of vasodilation.

Increased vascular tone induced by increased pressure or increased agonist addition has been shown to significantly reduce EDH‐induced vasodilation (Gschwend et al., 2003; Yarova et al., 2013). A suggested mechanism is the release of K^+^ from VSMC (Richards et al., 2001). When NE is added and VSMC depolarize the following Ca^2+^ influx activates BK_Ca_ channels and K^+^ efflux increases to dampen the vasoconstriction. This increase in extracellular K^+^ saturates K_IR_ channels and Na^+^/K^+^‐ATPases on the VSMC. A following release of endothelial derived K^+^ during EDH will now elicit a smaller EDH‐induced vasodilation.

Increased renal sympathetic nerve activity has been linked to hypertension. This is illustrated by the observations that renal denervation lowers hypertension both in animals (Lee and Walsh, 1983; Gattone et al., 1984) and humans (Chen and Upadhyay, 2017). Increased renal sympathetic nerve activity leads to constriction of renal resistance vessels. In 12‐week‐old SHR renal denervation not only reduced blood pressure but also led to increased afferent arteriolar diameter after 2 weeks (Gattone et al., 1984). Renal vasoconstriction in response to the sympathetic neurotransmitter norepinephrine (NE) is to a large extent dependent on activation of voltage operated Ca^2+^ channels (VOCC) (Salomonsson and Arendshorst, 1999). This indicates that NE induces a depolarization in renal VSMC similar to what is found in other vascular beds. It has been shown that NE induces a larger depolarization and a stronger contraction in VSMC from tail arteries from SHR compared to WKY (Hermsmeyer, 1976). Also, mesenteric VSMC from SHR have a higher intracellular Ca^2+^ concentration (Touyz and Schiffrin, 1997) and a higher expression of L‐type VOCC (Pratt et al., 2002) compared to normotensive rats. In afferent arterioles, NE increased the Ca^2+^ sensitivity to increase Ang II‐induced vasoconstriction (Lai et al., 2009). Thus, a connection between increased sympathetic activity and increased VSMC contractility seems likely.

The aim of the present study was to investigate the EDH‐induced vasodilation in the kidney and the potential physiological role thereof in the renal microcirculation in hypertensive and normotensive rats in vivo. The main hypothesis was that the increased renal vascular resistance (RVR) found in SHR would reduce the EDH response in vivo. The increased RVR could be caused by the increase in *V*_m_ and/or intracellular Ca^2+^ found in VSMC from SHR. The increased RVR could be induced by increasing blood pressure or increased sympathetic activity. Thus, the magnitude of the renal EDH response may change in hypertensive rats with age and development of hypertension as a consequence of the increased blood pressure per se or as an effect of increased activation of adrenergic receptors caused by increased renal sympathetic activity seen in hypertension (Lundin et al., 1984).

Experiments were performed in normotensive C57BL/6 mice, Sprague–Dawley (SD) rats, and SHR. SD rats were chosen because the WKY, normally used as control for SHR, has been shown to have abnormal Ca^2+^ sensitivity and connexin expression in resistance vessels compared to normotensive outbred Wistar and SD rats (Mulvany and Korsgaard, 1983; Braunstein et al., 2009). In isolated renal interlobar arteries from normotensive mice and rats, we tested if increased preconstriction (mimicking increased RVR) in general would affect renal EDH‐induced vasodilation. In anesthetized SD and SHR we examined the effect of intrarenal bolus infusion of ACh on renal blood flow (RBF). The infusions were done before and after treatment with the NO synthase inhibitor N(ω)‐nitro‐L‐arginine methyl ester (L‐NAME) and the cyclooxygenase inhibitor indomethacin. Thereafter we assessed the effect of reducing renal vascular resistance using prazosin (an *α*_1_‐adrenergic receptor antagonist reducing activation of adrenergic receptors) or papaverine (a phosphodiesterase inhibitor). Increases in renal vascular resistance were performed using NE (to increase activation of adrenergic receptors) or angiotensin II.

## Methods

The in vivo experiments were performed on 27 normotensive Sprague–Dawley (SD) rats and 30 SHR weighing 298 ± 5 g and 292 ± 4 g, respectively (app. 12 weeks old). The animals were purchased from Taconic (Denmark and USA). All experiments were approved by the Danish National Animal Experiments Inspectorate and conform to the European Convention for the Protection of Vertebrate Animals used for Experimental and other Scientific Purposes. The in vitro studies were performed on 10 SD rats and four C57/Bl6 mice weighing 303 ± 4 g and 29 ± 1 g, respectively. These animals were also purchased from Taconic (Denmark). Animals were held in the animal facility at University of Copenhagen with a 12:12 h light/dark cycle. Animals had free access to drinking water and standard diet. All procedures involving animals were in accordance with the ethical standards of the University of Copenhagen at which the studies were conducted.

### EDH in isolated renal interlobar arteries

Rats were anaesthetized with 5% isofluran delivered in 35% oxygen and 65% nitrogen and euthanized with spinal cord dislocation. Mice were euthanized with spinal cord dislocation. The kidneys were excised and bathed in cold dissection buffer (in mmol/L: NaCl 135, KCl 5, MgSO_4_·7H_2_O 1, HEPES 10, glucose 5, CaCl_2_ 1 and albumin 5 g/L, pH 7.4). Renal interlobar arteries were dissected and cut into 2‐mm pieces. The arteries were threaded onto two stainless steel wires (Ø40 *µ*m) and transferred to a preheated (37°C) myograph chamber (Danish Myograph Technology A/S, Aarhus, Denmark) containing a physiological saline solution (PSS in mmol/L: NaCl 130, NaHCO_3_ 14.9, KCl 4.7, MgSO_4_·7H_2_O 1.17, KH_2_PO_4_ 1.18, glucose 5.5, CaCl_2_ 1.6, EDTA 0.026) aerated with 95% O_2_ and 5% CO_2_, resulting in a pH of 7.4. The vessels were normalized to a tension equivalent to 0.9 times the tension found at a transmural pressure equivalent to 100 mmHg according to a previous protocol (Mulvany and Halpern, 1977).

#### Myograph protocol

All protocols were initiated with two exposures to PSS containing 60‐mmol/L K^+^ and 10‐*µ*mol/L norepinephrine (NE) serving as viability test and reference contractions. Vessels developing a tension <1 mN (rats) or <0.75 mN (mice) were discarded. The vessels were incubated for 30 min with PSS containing L‐NAME (300 *µ*mol/L) and indomethacin (3 *µ*mol/L) to inhibit production of NO and PGI_2_. The vessels were then preconstricted with increasing concentrations of NE (10–500 nmol/L; rats: *n* = 7; mice: *n* = 4) or increasing concentrations of U46619 (10–500 nmol/L; rats: *n* = 6). When a stable tension was achived (~3 min) ACh (10 *µ*mol/L) was added to the chamber. For each vasoconstrictor concentration the reduction in tension in response to ACh was assessed.

### Renal EDH in normo‐ and hypertensive rats in vivo

Rats were surgically prepared as previously described (Frandsen et al., 2016). Briefly, rats were anesthetized with 8% sevoflurane. In the left jugular vein, two catheters were inserted for continuous infusion of a muscle relaxant, cisatracurium (0.6 mg/mL), and isotonic saline (both 1.2 mL/h). The catheters were also used for i.v. administration of a nonselective inhibitor of COX 1 and 2, Indomethacin (2 mg/mL, 144 *µ*L/min for 5 min) and an inhibitor of NO synthesis, L‐NAME (2.5 mg/mL, 20 *µ*L/min for 30 min). A catheter filled with heparinized saline was inserted in the right carotid artery for continuous monitoring of the arterial blood pressure. The trachea was intubated and the rat was moved to a thermostatically controlled heating plate that maintained body temperature at ~37°C and was connected to a respirator (~65 breaths/min; tidal volume 8 mL/kg). Incisions were made to expose the renal and the femoral artery. In order to infuse drugs into the left renal artery, a catheter (PE‐10) was inserted in the left femoral artery, from where it was advanced into the renal artery. An ultrasonic flow probe (Transonic, lPR) was placed around the left renal artery to allow continuous monitoring of RBF. The left ureter was catheterized (PE‐10 connected to PE‐50) to ensure free urine flow. After the surgical procedure was completed, the rats recovered for ~30 min before the experimental protocol was initiated.

#### Experimental protocols

##### Reduced vasoconstriction

Isotonic saline was infused for 5 min (144 *µ*L/min) into the renal artery to obtain baseline RBF and accommodate to intrarenal infusions. Hereafter ACh (0.5 *µ*mol/L) was infused for 1.5 min. Next, indomethacin and L‐NAME was given intravenously. After 30 min, isotonic saline was infused intrarenally for 5 min followed by ACh for 1.5 min. The *α*_1_‐adrenergic antagonist prazosin (10 *µ*mol/L) or the phosphodiesterase inhibitor papaverine (6.25 *µ*mol/L) was then infused for 5 min into the renal artery followed by ACh for 1.5 min.

##### Increased vasoconstriction

A 3‐min intrarenal saline infusion (144 *µ*L/min) preceded the 1.5 min ACh infusion. The ACh response was assessed before and after intrarenal infusion of NE (0.0279 nmol/L) or Ang II (0.0665 nmol/L). Thereafter, indomethacin and L‐NAME was administrated as described above and the protocol was repeated.

#### Drug concentrations

The drug concentration of the intrarenal infusions represents the estimated plasma concentration, at an estimated renal plasma flow (RPF) of 3 mL/min and an infusion rate of 144 *µ*L/min. The concentrations of prazosin (10 *µ*mol/L), papaverine (6.25 *µ*mol/L), NE (0.0279 nmol/L) and Ang II (0.0665 nmol/L) are based on preliminary experiments. The chosen dosages induced a slight change in RBF (or MAP) during the 5 or 3 min pretreatment without changing RBF significantly.

### Calculations and statistical analysis

Relative relaxation in isolated interlobar arteries was calculated from the maximal tension elicited by NE or U46619 and the maximal dilatation elicited by ACh.

Values for MAP and RBF were found as the mean of the last 30 sec before initiating ACh infusion and the last 30 sec of the ACh infusion. Data are presented as the means ± SEM. Changes in baseline RBF and MAP (Tables 1, 2, 3, 4) were analyzed by One‐Way RM ANOVA using Student–Newman–Keuls (SNK) as post hoc test.

**Table 1:** MAP and RBF in SD (*n* = 8) and SHR (*n* = 8) before and after treatment with indomethacin and L‐NAME (Indo/L‐N) and after treatment with the *α*‐adrenergic receptor antagonist prazosin (Indo/L‐N/prazo).

	Saline	Indo/L‐N	Indo/L‐N/prazo
Sprague–Dawley			
MAP (mmHg)	97 ± 4	109 ± 8*	87 ± 7*, ‡
ACh MAP (mmHg)	95 ± 4	109 ± 8†	86 ± 7‡
RBF (mL/min)	6.9 ± 0.4	4.6 ± 0.4†	4.7 ± 0.5†
ACh RBF (mL/min)	8.7 ± 0.5	5.2 ± 0.4†	5.2 ± 0.5†
RVR (mmHg/mL/min)	14.5 ± 1.1	21.4 ± 2.9†	19.7 ± 2.6*, ‡
ACh RVR (mmHg/mL/min)	11.4 ± 1.0	18.2 ± 2.8†	17.6 ± 2.4†, ‡
SHR			
MAP (mmHg)	138 ± 4	183 ± 6†	163 ± 4†, ‡
ACh MAP (mmHg)	131 ± 4	182 ± 6†	158 ± 5†, ‡
RBF (mL/min)	5.1 ± 0.2	2.7 ± 0.3†	2.6 ± 0.2†
ACh RBF (mL/min)	7.7 ± 0.6	3.0 ± 0.3†	3.5 ± 0.4†
RVR (mmHg/mL/min)	27.2 ± 1.2	72.9 ± 6.8†	65.3 ± 5.2†
ACh RVR (mmHg/mL/min)	17.9 ± 1.4	66.6 ± 7.3†	49.0 ± 5.7†, ‡

ACh values, values measured after 90 sec ACh treatment.

* *P* < 0.05 versus saline.

^†^ *P* < 0.01 versus saline.

^‡^ *P* < 0.01 versus Indo/L‐N.

**Table 2:** MAP and RBF in SD (*n* = 8) and SHR (*n* = 8) before and after treatment with indomethacin and L‐NAME (Indo/L‐N) and after treatment with the phosphodiesterase inhibitor papaverine (Indo/L‐N/papa).

	Saline	Indo/L‐N	Indo/L‐N/papa
Sprague–Dawley			
MAP (mmHg)	106 ± 3	129 ± 6*	128 ± 6*
ACh MAP (mmHg)	105 ± 3	128 ± 5*	128 ± 6*
RBF (mL/min)	8.7 ± 0.5	5.9 ± 0.2*	6.6 ± 0.5*
ACh RBF (mL/min)	10.7 ± 0.6	6.5 ± 0.2*	6.7 ± 0.5*
RVR (mmHg/mL/min)	12.4 ± 0.6	22.1 ± 1.1*	20.6 ± 2.1*
ACh RVR (mmHg/mL/min)	9.9 ± 0.4	19.9 ± 1.1*	20.2 ± 2.2*
SHR			
MAP (mmHg)	141 ± 6	188 ± 7*	186 ± 7*
ACh MAP (mmHg)	136 ± 6	186 ± 6*	184 ± 7*
RBF (mL/min)	6.3 ± 0.6	3.4 ± 0.4*	4.2 ± 0.4*, †
ACh RBF (mL/min)	9.1 ± 0.6	3.8 ± 0.4*	4.1 ± 0.4*
RVR (mmHg/mL/min)	24.8 ± 3.6	64.1 ± 11.3*	48.5 ± 6.9*, †
ACh RVR (mmHg/mL/min)	15.7 ± 1.8	55.6 ± 9.2*	48.3 ± 5.9*

ACh values, values measured after 90‐sec ACh treatment.

* *P* < 0.01 versus saline.

^†^ *P* < 0.01 versus Indo/L‐N.

**Table 3:** MAP and RBF in SD (*n* = 5) and SHR (*n* = 8) before and after treatment with indomethacin and L‐NAME (Indo/L‐N) and after treatment with the adrenergic receptor agonist NE (Indo/L‐N/NE).

	Saline	Indo/L‐N	Indo/L‐N/NE
Sprague–Dawley			
MAP (mmHg)	110 ± 3	147 ± 3*	147 ± 2*
ACh MAP (mmHg)	109 ± 3	148 ± 3*	148 ± 3*
RBF (mL/min)	7.9 ± 0.3	4.4 ± 0.5*	4.0 ± 0.4*
ACh RBF (mL/min)	10.0 ± 0.6	4.8 ± 0.5*	4.7 ± 0.4*
RVR (mmHg /mL/min)	14.0 ± 0.7	36.2 ± 5.5*	39.7 ± 5.1*
ACh RVR (mmHg /mL/min)	10.9 ± 0.4	33.4 ± 4.7*	32.8 ± 3.6*
SHR			
MAP (mmHg)	153 ± 6	183 ± 6*	180 ± 8*
ACh MAP (mmHg)	147 ± 6	182 ± 7*	177 ± 8*
RBF (mL/min)	6.8 ± 0.8	3.7 ± 0.5*	3.2 ± 0.4*
ACh RBF (mL/min)	9.0 ± 0.8	4.2 ± 0.4*	4.1 ± 0.5*
RVR (mmHg /mL/min)	24.3 ± 2.7	56.8 ± 8.3*	63.7 ± 8.5*
ACh RVR (mmHg /mL/min)	17.1 ± 1.5	46.3 ± 4.0*	47.1 ± 5.4*

ACh values, values measured after 90‐sec ACh treatment.

* *P* < 0.01 versus saline.

**Table 4:** MAP and RBF in SD (*n* = 6) and SHR (*n* = 6) before and after treatment with indomethacin and L‐NAME (Indo/L‐N) and after treatment with angiotensin II (Indo/L‐N/AngII).

	Saline	Indo/L‐N	Indo/L‐N/AngII
Sprague–Dawley			
MAP (mmHg)	97 ± 2	125 ± 7*	125 ± 8*
ACh MAP (mmHg)	96 ± 3	125 ± 7*	124 ± 8*
RBF (mL/min)	7.5 ± 0.8	5.8 ± 0.6*	5.6 ± 0.7*
ACh RBF (mL/min)	10.1 ± 1.2	6.7 ± 0.7*	6.7 ± 0.8*
RVR (mmHg/mL/min)	13.9 ± 1.7	23.6 ± 3.7*	25.0 ± 4.7*
ACh RVR (mmHg/mL/min)	10.5 ± 1.5	20.5 ± 3.5*	20.8 ± 4.0*
SHR			
MAP (mmHg)	171 ± 6	202 ± 2*	195 ± 3*
ACh MAP (mmHg)	166 ± 6	200 ± 3*	194 ± 3*
RBF (mL/min)	6.6 ± 0.4	3.4 ± 0.5*	3.0 ± 0.4*
ACh RBF (mL/min)	8.4 ± 0.4	4.0 ± 0.5*	3.7 ± 0.4*
RVR (mmHg /mL/min)	26.7 ± 1.9	73.0 ± 17.3*	72.9 ± 11.0*
ACh RVR (mmHg /mL/min)	19.8 ± 0.9	56.6 ± 9.8*	57.1 ± 7.6*

ACh values, values measured after 90‐sec ACh treatment.

* *P* < 0.01 versus saline.

**Figure 1:**
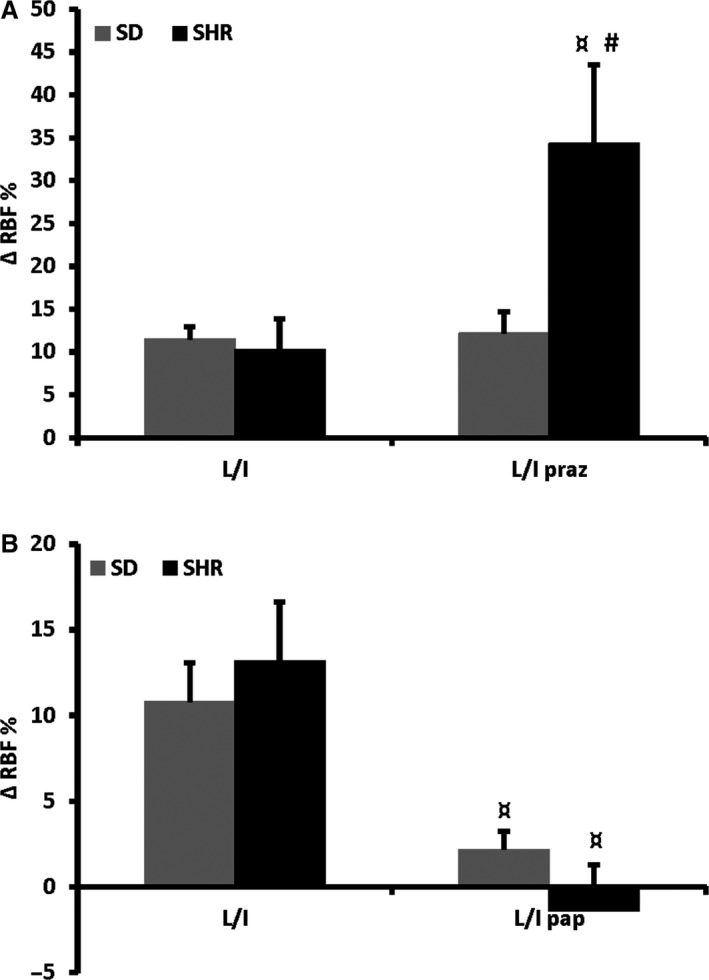
Increases in RBF during intrarenal infusion of ACh compared to baseline. All experiments were done in the presence of L‐NAME and indomethacin (L/I). After pretreatment with prazosin the response was significantly increased in SHR (*n* = 8) but not in SD (*n* = 8) (A). After pretreatment with papaverine the response was significantly reduced in both strains (SD; *n* = 8, SHR *n* = 8) (B). *P < 0.01 versus SD; ^†^ *P* < 0.01 versus L/I.

**Figure 2:**
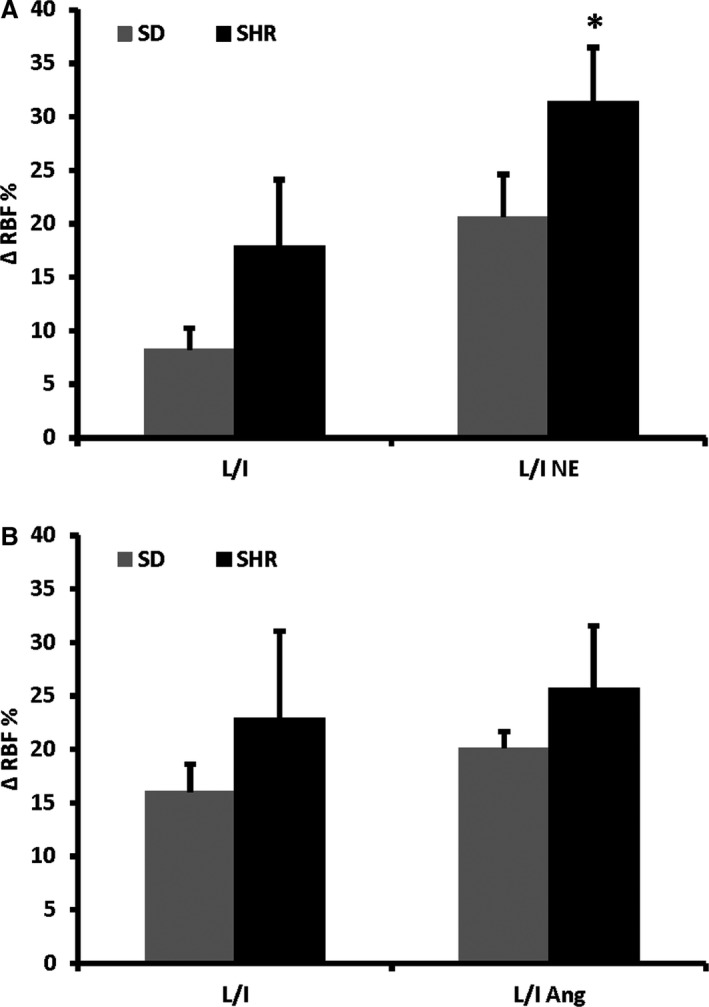
Increases in RBF during intrarenal infusion of ACh compared to baseline. All experiments were performed in the presence of L‐NAME and indomethacin (L/I). After pretreatment with NE the renal EDH response increased in SHR (*n* = 8) but not in SD (*n* = 5) (A). After pretreatment with Ang II the EDH response was unchanged in both strains (SD; *n* = 6, SHR *n* = 6) (B). *P < 0.05 versus L/I.

ACh‐induced changes in RBF was calculated as % change from baseline (100%) to account for the different baselines in SD and SHR and after treatment. Differences within and between groups (Figs. 1, 2, 4) were analyzed using a two‐way ANOVA followed by SNK. P‐values less than 0.05 were considered statistical significant.

## Results

### EDH in isolated renal interlobar arteries from rats and mice

**Figure 3:**
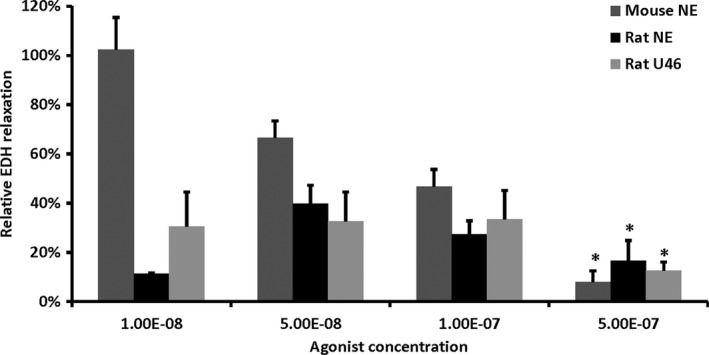
Increased preconstriction using NE or U46619 reduced the renal vasodilation elicited by 10‐*µ*mol/L ACh after treatment with L‐NAME and indomethacin. Rat vessels responded poorly to the lowest agonist concentration (*n* = 3 for NE and *n* = 2 for U46619). The EDH response was not different between species (mice; *n* = 4, rats; *n* = 10). *P < 0.05 versus 50 nmol/L.

The vasoconstrictor response elicited by 10 nmol/L NE in rat interlobar arteries was very weak (<1 mN) so most vessels were excluded from the analysis of this NE dose. In interlobar arteries from mice and rats a decreased relaxation in response to 10 *µ*mol/L ACh in the presence of indomethacin and L‐NAME was found with increasing preconstriction from the adrenergic agonist NE (Fig. 3). In rats, a similar result was seen using the thromboxane A2 receptor agonist U46619 as a preconstrictor (Fig. 3).

### Renal EDH in normo‐ and hypertensive rats in vivo

Baseline values for MAP, RBF, and RVR are shown in Tables 1, 2, 3, 4 for both rat strains. As expected, baseline RBF was higher and MAP and RVR were lower in SD compared to SHR both before and after inhibition of NO and prostaglandin production showing that the SHR has increased renal vascular resistance compared to SD. Treatment with indomethacin and L‐NAME (Indo/L‐N) significantly increased MAP and reduced RBF in both strains. A 5‐min intrarenal pretreatment with the *α*_1_‐adrenergic antagonist prazosin significantly reduced MAP and RVR in both SD and SHR but did not affect RBF (Table 1). Pretreatment with the nonspecific phosphodiesterase inhibitor papaverine increased RBF and reduced RVR significantly in SHR but had no effect on MAP in either strain (Table 2). A 3‐minute intrarenal pretreatment with either NE or Ang II had no significant effect on baseline MAP, RBF and RVR but tended to decrease RBF in both strains (Tables 3 and 4).

**Figure 4:**
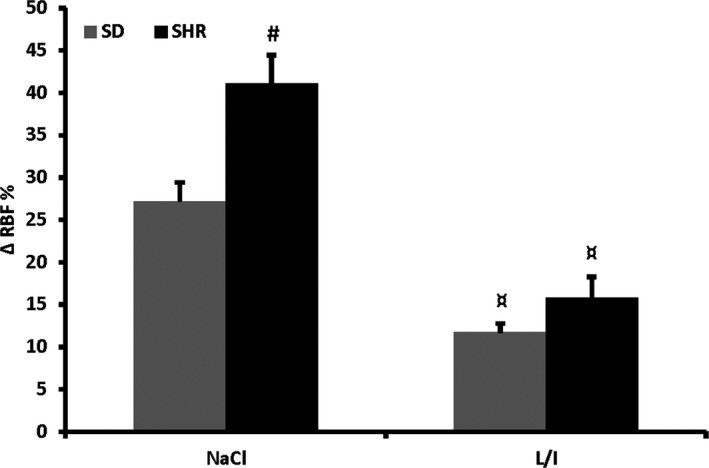
Increases in RBF compared to baseline during intrarenal infusion of ACh in SD (*n* = 27) and SHR (*n* = 30). The increase in RBF was significantly higher in SHR. After treatment with L‐NAME and indomethacin (L/I) the response was significantly lowered in both rat strains. The EDH response was not different between strains. * *P* < 0.01 versus SD; ^†^ *P* < 0.01 versus NaCl.

Data from all rats are combined in Figure 4 to show the effect of ACh on RBF before and after treatment with indomethacin and L‐NAME. The renal ACh response was significantly larger in SHR compared to SD. After treatment with L‐NAME and indomethacin the EDH‐induced vasodilation was similar between the strains. In both strains ACh elicited a significantly smaller increase in RBF after L‐NAME/Indo compared to before treatment.

#### Reduced renal vascular resistance

The renal EDH response was similar in SD and SHR (Fig. 1A and B). Following pretreatment with the *α*_1_‐adrenergic antagonist prazosin the renal EDH response in SHR increased significantly compared to the response in SD (Fig. 1A). Interestingly, pretreatment with the nonspecific phosphodiesterase inhibitor papaverine abolished the renal EDH‐response in both rat strains (Fig. 1B).

#### Increased renal vascular resistance

As observed before the renal EDH response was similar in SD and SHR (Fig. 2A and B). Following pretreatment with the adrenergic agonist NE the renal EDH response increased significantly in SHR (Fig. 2A). Pretreatment with Ang II did not affect the EDH response in neither strain (Fig. 2B).

## Discussion

The aim of the present study was to investigate the EDH‐induced vasodilation in the renal microcirculation in hypertensive and normotensive rats in vivo. Our main hypothesis was that the increase in RVR seen in hypertension, possibly induced by the increased blood pressure or increased sympathetic nerve activity, would reduce endothelium‐dependent vasodilation.

First, we examined the effect of preconstriction in larger conductance vessels in vitro. Increasing concentrations of NE or U46619 increased the tension elicited by interlobar arteries in vitro, most likely by an increased VSMC membrane depolarization (Salomonsson and Arendshorst, 1999). This significantly reduced renal EDH‐induced vasodilation in vitro. However, as similar results were obtained using the TxA_2_ analouge U46619, these results indicate that it is the increased tension *per se* and not an increased activation of adrenergic receptors that reduces EDH‐induced vasodilation under isometric conditions. We have previously shown that EDH‐induced vasodilation in interlobar arteries is initiated by activation of endothelial IK_Ca_ and SK_Ca_ channels releasing K^+^ to activate K_ir_ channels and Na^+^/K^+^‐APTases on the vascular smooth muscle cell (Rasmussen et al., 2016; Brasen et al., 2018). However, if the concentration of K^+^ surrounding the VSMC increases, the K_ir_ channels and Na^+^/K^+^‐APTases become saturated and EDH‐induced vasodilation decreases (Edwards and Weston, 2004) as also seen in the present study.

In isolated renal arteries from 8‐week‐old SHR the EDH‐induced vasodilation is increased compared to age‐matched WKY (Bussemaker et al., 2003). However, at the age of 22 months the renal EDH response in SHR is abolished (Bussemaker et al., 2003) indicating that with increasing age and blood pressure EDH diminishes. Measurements of resting membrane potentials from VSMC from renal arteries show a significant depolarization at increasing age in SHR but not in WKY (Bussemaker et al., 2003). The hyperpolarizing effect of renal EDH on VSMC is also significantly decreased in 22‐month‐old SHR. At 8 months of age the renal EDH‐induced vasodilation measured in the renal artery is significantly reduced in SHR compared to WKY (Michel et al., 2008). In 26‐week‐old SHR EDH‐induced vasodilation measured at increasing doses of ACh in renal segmental arteries is right‐shifted compared to the curve measured in renal arteries from WKY but the maximum vasodilation at 1‐*µ*mol/L ACh is identical (Dohi et al., 1996). Thus, is seems as if renal EDH is present in younger SHR but diminishes at increasing age and blood pressure.

The results from the isolated interlobar arteries are based on isometric constriction. It has been reported that EDH‐induced vasodilation behaves differently depending on the experimental set‐up. In isolated A. gracilis from mice ACh‐induced vasodilation during isometric conditions was significantly smaller after inhibition of L‐NAME and indomethacin compared to before (Boettcher and Wit, 2011). However, during isobaric conditions and in vivo the ACh‐induced vasodilation before and after inhibition of eNOS and COX was similar indicating that EDH is the primary endothelial vasodilator during these conditions. This is in contrast to the renal EDH where inhibition of eNOS and COX significantly reduced the Ach‐induced vasodilation both in vitro and in vivo (Majid and Navar, 1992; Dautzenberg and Just, 2013; Rasmussen et al., 2016). The renal in vitro results are from larger conductance vessels whereas the in vivo measurements show the integrated responses from small resistance vessels. EDH has been shown to be more pronounced in smaller conductance vessels compared to larger conductance vessels (Boettcher and Wit, 2011) which may further complicate the picture.

Secondly, we characterized EDH in SD and SHR in vivo, and studied the effects of sympathetic activity and increased vascular resistance on EDH. In the present study we found that in anesthetized normotensive SD rats and SHR increases in RBF were elicited by intrarenal infusions of 0.5‐*µ*mol/L ACh. In SHR the renal ACh‐induced vasodilation was significantly larger compared to the normotensive rats. This suggests that at 12 weeks of age renal endothelial dysfunction is not prominent in SHR. After inhibition of NO and PGI_2_ production the ACh‐induced renal vasodilation was similar between the two strains and significantly smaller than before inhibition indicating an increased importance of NO in 12‐week‐old SHR. This corresponds well with our observed increased effect of L‐NAME on RVR in SHR compared to SD (Tables 1, 2, 3, 4). In Wistar rats the renal EDH response to bolus infusion of ACh has been investigated in vivo (Vriese et al., 2002; Edgley et al., 2008). Here it was also found that in normotensive rats in vivo, inhibition of eNOS and COX significantly reduces the vasodilatory response to ACh. This is in contrast to the above mentioned results from Boettcher et al. (Boettcher and Wit, 2011) but could possibly be explained by the use of different species and different vascular beds being investigated.

To test the hypothesis that an increased renal vascular resistance also would affect the EDH‐induced renal vasodilation in vivo we treated the rats with vasodilators (prazosin and papaverine) and vasoconstrictors (NE and Ang II). Papaverine and Ang II were chosen to reduce and increase RVR by other means than through *α*‐adrenergic receptors. Treatment with the *α*_1_‐adrenergic antagonist prazosin reduced MAP compared to L‐NAME/indomethacin but did not significantly increase RBF. However, the renal vascular resistance decreased significantly in both strains. Assessment of the EDH‐induced renal vasodilation showed that prazosin significantly increased the EDH response in SHR but not in SD. These results seem to correspond to previous findings showing that VSMC from SHR are more depolarized and constrict more strongly to NE (Hermsmeyer, 1976). Inhibition of the increased vasoconstriction from NE using prazosin could reduce the depolarization of the VSMC in SHR. Thus, it may be easier to induce an EDH‐induced vasodilation after prazosin in SHR.

When reducing renal vascular resistance using papaverine we found that the EDH‐induced renal vasodilation was absent in both strains. Papaverine did not change MAP in either strain and only increased RBF significantly in SHR. Interestingly, the use of papaverine seemingly abolished the ability of EDH to dilate the renal vasculature in both strains. This could suggest that the renal vasculature is not able to dilate any further but the dose of papaverine used in our study only increased RBF slightly suggesting that the renal vasculature is not maximally dilated. In dogs papaverine completely abolished renal autoregulation in contrast to nitro‐compounds such as sodium nitroprusside (Ogawa and Ono, 1986) which could suggest that the renal vasculature loses the ability to regulate tone after papaverine treatment. Papaverine increases cAMP and cGMP in VSMC from renal arteries (Karsten et al., 2003) leading to decreases in intracellular [Ca^2+^] (Gagnon et al., 1980). However, in rabbit iliac arteries the EDH response is potentiated by increasing cAMP in vascular smooth muscle cells (Griffith et al., 2002). Another explanation could therefore be that the concentration of cAMP in VSMC is already maximal and no further increase is generated when ACh is added thus eliminating the EDH response. It has also been suggested that the renal EDH is either K^+^ released from EC (Edgley et al., 2008; Rasmussen et al., 2016) or transfer of the hyperpolarization through gap junctions (Vriese et al., 2002). Inhibition of renal gap junctions did not affect the renal vasodilation induced by papaverine suggesting that there is no interaction between papaverine and gap junctions in the renal vasculature (Vriese et al., 2002). However, several K^+^ channels are activated by protein kinase A and G (Salomonsson et al., 2017). Possibly, the release of K^+^ from VSMC is high enough to saturate the K_ir_ channels and Na^+^/K^+^‐ATPases and prevent a further hyperpolarization in this setting.

Pretreatment with the vasoconstrictors NE and Ang II induced a slight reduction in RBF in both strains, but the effect on RVR was not significant in comparison to the RVR measured after L‐NAME/indomethacin treatment. However, due to the relative long half‐life of L‐NAME in plasma (Conner et al., 2000) the experiments using pretreatment with vasodilators or inhibitors were always carried out as the last experiment. As the sevoflurane anesthesia will slowly reduce MAP and RBF it is possible that the effect of the pretreatment is underestimated in our experiments. When comparing the RVR found after NE treatment in SD (39.7‐mmHg/mL/min) with the RVR measured directly before NE infusion (35.7‐mmHg/mL/min) the effect is significant.

The pretreatment with NE did however increase the EDH‐induced vasodilation observed in SHR which is the same effect as seen when reducing RVR. Considering that the VSMC from SHR are proposed to be more sensitive to NE (Mulvany et al., 1980), NE would theoretically induce a stronger depolarization and decrease the EDH‐induced vasodilation. The present increase in renal EDH‐induced vasodilation in vivo is thus hard to explain. However, vasoconstriction elicited by an *α*_1_‐adrenergic receptor agonist (phenylephrine) is time dependently reduced in rat mesenteric arteries (Jin et al., 2011). This reduction is abolished by removal of the endothelium but not by treatment with indomethacin and L‐NAME indicating that *α*_1_‐adrenergic stimulation increases EDH production. Possibly the increased depolarization and contraction observed in VSMC from SHR in response to NE could be caused by an increased expression of *α*_1_‐adrenergic receptors in SHR. Studies show than in VSMC from aorta there seems to be an increased expression of *α*_1A_‐ and *α*_1B_‐receptors compared to WKY but this was not found in carotid arteries (Edith‐Rodriguez et al., 2013). Increased function of *α*_1A_‐ and *α*_1D_‐receptors in the hindlimb vascular bed of SHR has also been found (Ye and Colquhoun, 1998).

In conclusion, we found that the renal EDH response measured in young SHR in vivo is not reduced compared to normotensive rats. Importantly, at this age renal endothelial dysfunction does not seem to be prevalent in SHR even though blood pressure is significantly increased and renal blood flow is significantly lower. This is in contrast to the EDH response found in isolated renal arteries from 8‐week‐old SHR where the EDH response was higher than in normotensive WKY. At 14 weeks of age the EDH‐induced vasodilation in afferent arterioles was significantly reduced. Possibly, the EDH response measured in our experiments at 12 weeks is in the transition from an increased to a decreased renal EDH response. The changes induced in RVR did not affect the renal EDH in the predicted direction. *α*1‐receptor inhibition with prazosin to reduce RVR enhanced EDH in SHR but not in SD in vivo. *α*1‐receptor activation with NE causing an increased RVR also enhanced EDH in SHR in vivo. Thus, both increases and decreases in RVR elicited a larger EDH‐induced vasodilation in SHR and had no effect in normotensive rats. Possibly, the induced changes are buffered in the in viv*o* setting but not in vitro.

## Conflict of Interest

None.
